# Manufacture and evaluation of a HER2-positive breast cancer immunotoxin 4D5Fv-PE25

**DOI:** 10.1186/s12934-023-02115-0

**Published:** 2023-05-17

**Authors:** Yanjie Peng, Zhengli Wu, Zheng Pang, Lin Zhang, Dandan Song, Fang Liu, Yanhong Li, Tongjun Lin

**Affiliations:** 1 Clinical Medical Research Center for Women and Children Diseases, Shandong Provincial Maternal and Child Health Care Hospital Affiliated to Qingdao University, Jinan, 250014 China; 2 Panacea Bioscience inc, Halifax, NS Canada; 3 College of Fisheries, Southwest University, Beibei, Chongqing, 400715 China; 4 Innovative Institute of Chinese Medicine and Pharmacy, Shandong University of Traditional Chinese Medicine, Jinan, 250300 China

**Keywords:** Prokaryotic expression, Immunotoxin, HER2, Breast cancer, Manufacture, Evaluation

## Abstract

**Background:**

Human epidermal growth factor receptor 2 (HER2) positive breast cancer is an aggressive subtype, accounting for around 20% of all breast cancers. The development of HER2-targeted therapy has substantially improved patient outcomes. Nevertheless, the increasing rate of side effects and resistance to targeted drugs limit their efficacy in clinical practice. In this study, we designed and synthesized a new immunotoxin, 4D5Fv-PE25, which targets HER2-positive breast cancer, and evaluated its effectiveness in vitro and in vivo.

**Results:**

The 4D5Fv-PE25 was expressed in high-density *Escherichia coli* (*E. coli.*) using the fermentor method and refined via hydrophobicity, ion exchange, and filtration chromatography, achieving a 56.06% recovery rate. Additionally, the semi-manufactured product with 96% purity was prepared into freeze-dried powder by the lyophilized process. Flow cytometry was used to detect the expression of HER2 in SK-BR-3, BT-474, MDA-MB-231, and MDA-MB-468 breast cancer cell lines. The 3-(4,5-dimethylthiazol-2-yl)-2,5-diphenyl tetrazolium bromide (MTT) method was used for cytotoxicity assay, and the half-maximal inhibitory concentration (IC_50_) of 4D5Fv-PE25 lyophilized products to HER2-positive cell line SK-BR-3 was 12.53 ng/mL. The 4D5Fv-PE25 was injected into xenograft tumor mice via the tail vein on the 1st, 4th, and 8th day, it indicated that the growth of tumor volume was effectively inhibited for 24 days, although the 4D5Fv-PE25 was metabolized within 60 min by measuring the release of 3 H-Thymidine radiation.

**Conclusion:**

we succeeded in producing the 4D5Fv-PE25 freeze-dried powder using the prokaryotic expression method, and it could be employed as a potential drug for treating HER2-positive breast cancer.

**Graphical abstract:**

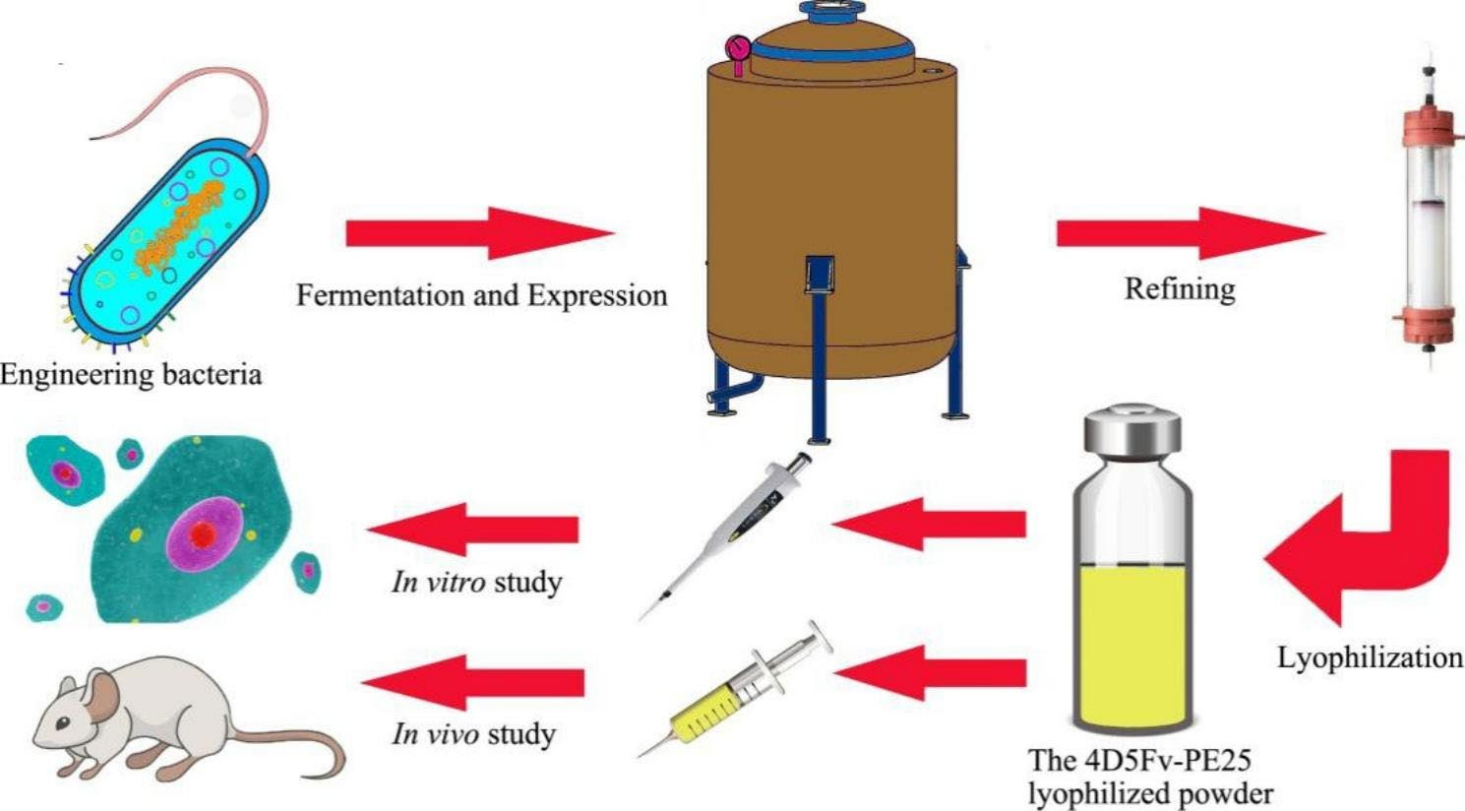

## Background

Breast cancer is the most prevalent neoplasm and a significant contributor to cancer-related deaths in women [1]. Based on immunohistochemistry, breast cancer is classified as hormone receptor positive (HR+), human epidermal growth factor receptor 2 positive (HER2+), and triple-negative (TNBC) [2]. Approximately 15-20% of all breast cancer cases are HER2-positive [3]. HER2-positive breast cancer is a subtype featured by poor differentiation, strong invasiveness, high recurrence and metastasis rate, short survival time, and unfavorable clinical outcomes [4]. HER2 overexpression in HER2-positive breast cancer is compared to normal cells, and it triggers a variety of downstream pathways, leading to increased proliferation of cancer cells. Therefore, HER2 is a valuable and rational therapeutic target for the treatment of HER2-positive breast cancer [5].

In the first-line category of drugs approved for treating HER2-positive breast cancer, trastuzumab is the gold standard. Small molecule drugs such as Lapatinib (a protein tyrosine kinase inhibitor for HER2-positive breast cancer) and Antibody-drug conjugates (ADCs) like trastuzumab emtansine (T-DM1) and trastuzumab deruxtecan (T-DXd) belong to the second-line drugs [5–7]. The ADCs is composed of an antigen-specific mAb conjugated to a potent cytotoxic agent for the treatment of HER2-positive breast cancer [5, 8]. However, the resistance rate of trastuzumab in HER2-positive breast cancer is 66–88% when used as a single agent and 20–50% when combined with chemotherapy [9]. Large molecular size inhibits the penetration of ADCs and monoclonal antibodies into solid tumors [10]. The preparation of ADCs requires the connection of trastuzumab with a potent cytotoxic agent, and the process has low efficiency with a risk of off-target toxicity and heterogeneous conjugates, resulting in a narrow therapeutic window. Small molecule drugs impair cancer cells’ growth by blocking signal transmission without completely killing tumor cells and are mostly used in drug-combination schemes [11]. Although drug resistance can be overcome by combining ADCs with other therapeutic drugs or improving the active structure of ADCs, there are still issues with the preparation of ADCs and the convenience of combining drugs [5]. Hence, there is a pressing need to develop more effective drugs and treatment regimens for managing HER2-positive breast cancer.

Immunotoxin is a heterogenous molecule that exerts specific targeted killing effects, consisting of tumor-specific recognition antibodies and toxin molecules. Compared to conventional drugs, it is better suited due to its strong effect and diminished incidence of drug resistance [12, 13]. Immunotoxin’s molecular mass is about one-third that of a monoclonal antibody, which makes it more penetrable into solid tumors. Immunotoxin may be generated in prokaryotic cells with high controllability, good preparation efficiency, and low preparation-quality risk [14]. Moxetumomab pasudotox is an approval-grade immunotoxin for the management of hairy cell leukemia, consisting of an anti-CD22 fragment and a 38 kDa PE toxin (a fragment of the *Pseudomonas* exotoxin A), approved by the Food and Drug Administration (FDA) [15]. As such, immunotoxins are a promising drug category.

In this research, a novel immunotoxin, 4D5Fv-PE25, was developed for HER2-positive breast cancer management. We evaluated the pharmaceutical properties of 4D5Fv-PE25 via cell viability assays and mice experiments. The findings demonstrated the capacity of 4D5Fv-PE25 to recognize and inhibit HER2 targets effectively in vivo and in vivo, making it a highly suitable candidate drug for the management of HER2-positive breast cancer.

## Results

### Design of the immunotoxins

**Fig. 1:**
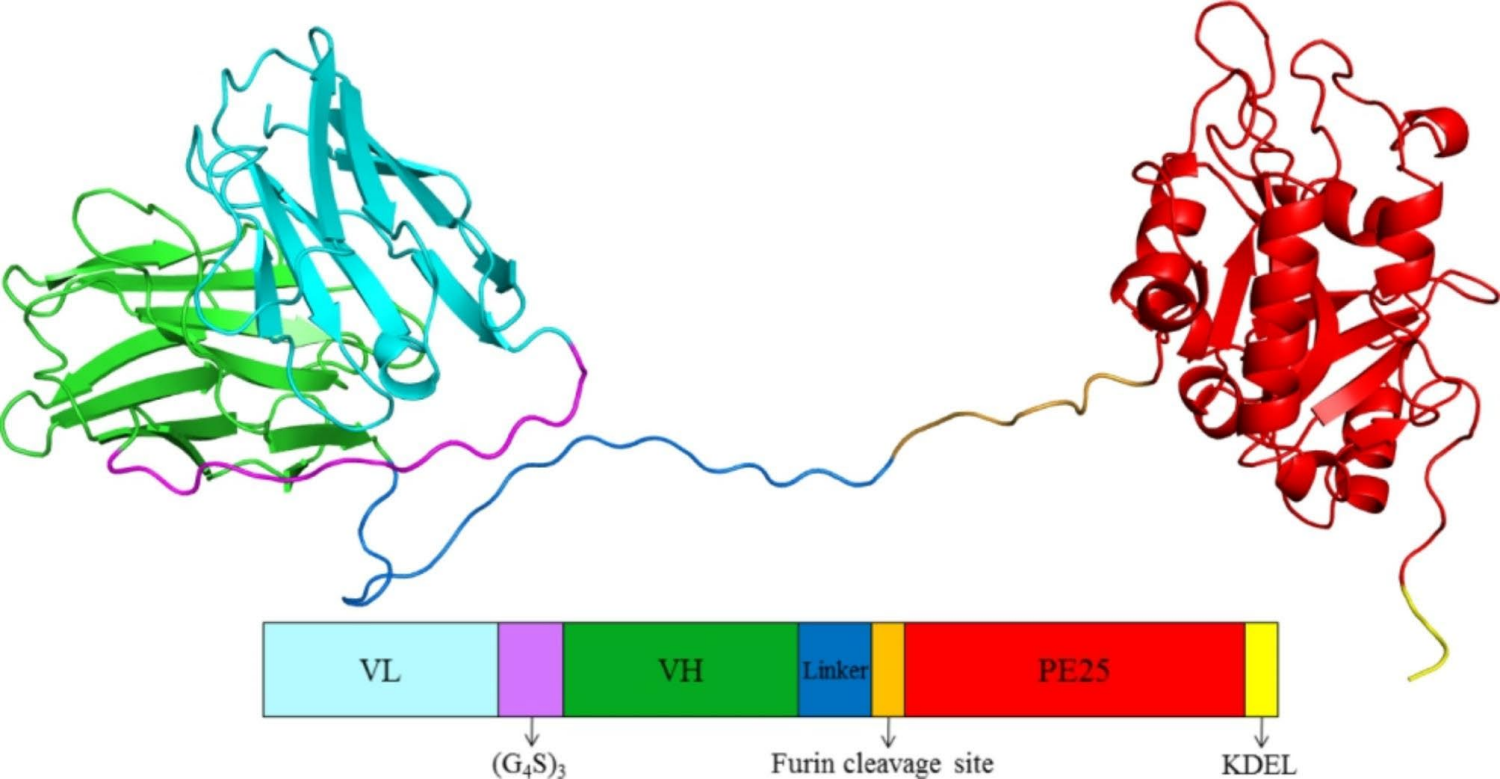
Structural model of the 4D5Fv-PE25. VL and VH: the variable region of trastuzumab, PE25: *Pseudomonas* exotoxin, KDEL: potent toxin fragment, G: glycine, S: serine, Linker: the last 20 amino acids of human muscle aldolase, characterized by flexibility, easy folding, and no antigenicity. The cartoon was generated with PyMOL software, in which the structure of VL, VH, and PE25 was built based on the X-ray crystal structure of HER2-binding scFv-Fab fusion (Protein Data Bank code: 6ZQK) and *Pseudomonas Aeruginosa* Exotoxin A (Protein Data Bank code: 6EDG).

The 4D5Fv-PE25 consists of the single-chain variable region of trastuzumab (VL and VH connected through (G_4_S)_3_) and a truncated form of *Pseudomonas* exotoxin A (PE25) (Fig. 1). The variable region of trastuzumab was linked to the PE25 sequence by the last 20 amino acids of human muscle aldolase and the furin cleavage site. The use of human muscle aldolase sequence as a linker helps to form the spatial conformation of the fusion protein [16], while the furin cleavage site ensures the safe release of PE25 in cancer cells. The PE25 part of the fusion protein is mutated at eight amino acid sites to reduce its immunogenicity [17]. The KDEL is a reserved sequence of the endoplasmic reticulum, which can make PE25 persistent inactivate protein elongation factor 2 and cause cell death by inhibiting protein synthesis [18]. The antibody part of the fusion protein binds to the HER2 receptor on the surface of cancer cells, the complex is internalized, and the PE25 part is released into the cytoplasm under the effect of enzymatic hydrolysis and kills the tumor cells [16, 19–21], where it inhibits protein synthesis and induces cell death. The 4D5Fv-PE25 has several advantages, such as a small molecular weight, high tumor penetration, and low immunogenicity, making it a promising candidate for targeted cancer therapy [22, 23].

### Preparation of inclusion bodies

**Fig. 2:**
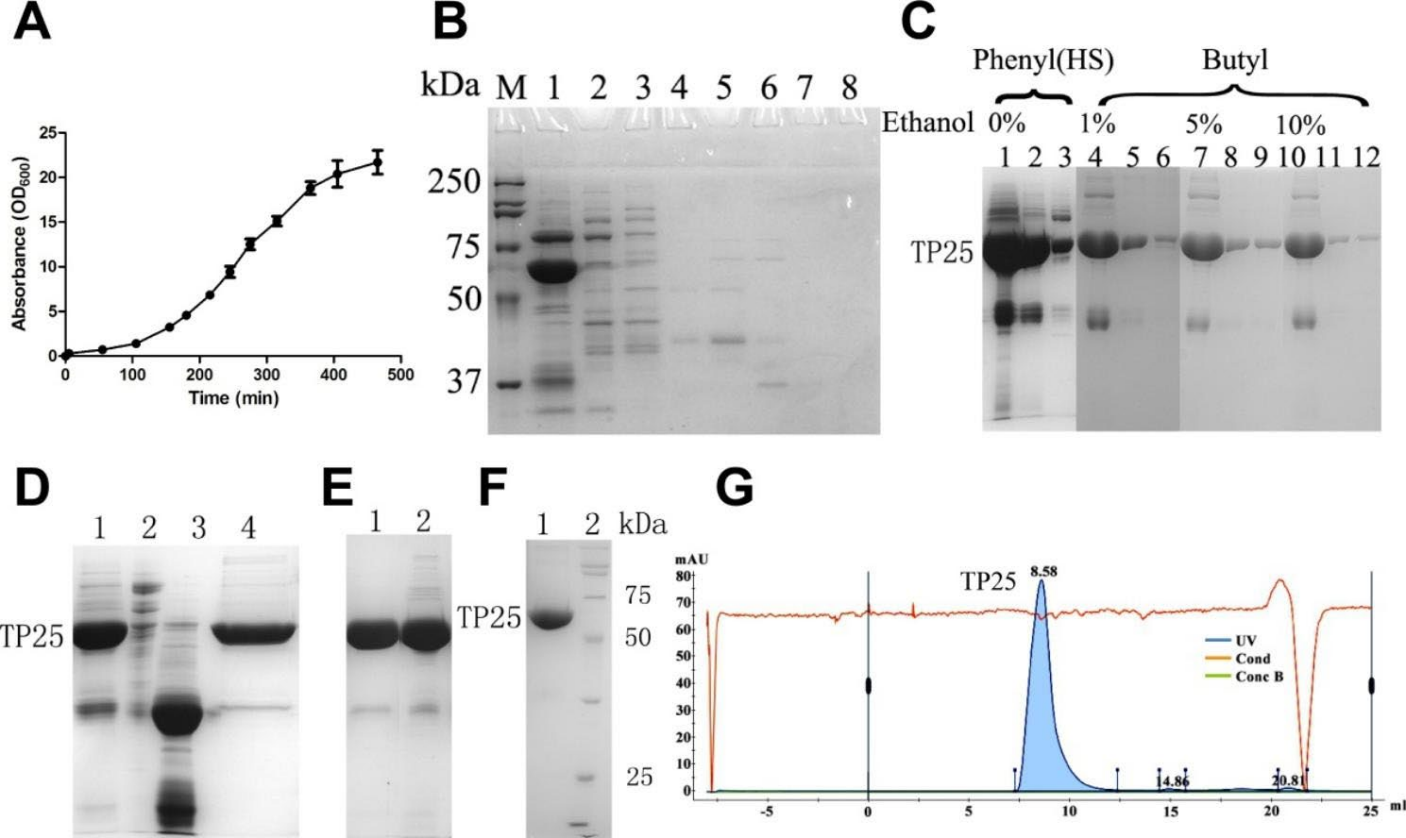
Growth curve of engineering bacteria and purity test of 4D5Fv-PE25. **A**: Growth curve of engineering bacteria (n = 3). **B**: Detection of the effect of removing impure protein by equilibrium buffer in Capto Butyl gel. M: marker, 1–8: 8 kinds of equilibrium buffer (30% saturation ammonium sulfate/chromatography buffer A (v/v)): 10%, 20%, 30%, 40%, 50%, 60%, 70%, 100%. **C**: Elution effect of different gels and eluent. 1: ethanol-free chromatography buffer A, 2: deionized water, 3: 0.5 M sodium hydroxide, 4: chromatography buffer A containing 1% ethanol (v/v), 5: deionized water, 6: 0.5 M sodium hydroxide, 7: chromatography buffer A containing 5% ethanol (v/v), 8: deionized water, 9: 0.5 M sodium hydroxide, 10: chromatography buffer A containing 10% ethanol (v/v), 11: deionized water, 12: 0.5 M sodium hydroxide. **D**: Purification effect of Capto S ImpAct and Capto Q. 1: the flowing through protein of Capto S Impact, 2: the binding protein of Capto S Impact, 3: the flowing through protein of Capto Q, 4: the binding protein of Capto Q. **E**: Purification effect of Capto S ImpAct at different flow rates. 1: 6.0 mm/min, 2: 15.0 mm/min. **F**: purity SDS-PAGE detection. **G**: Purity chromatographic detection

The *E. coli.* BL21 strain used in this study was engineered to overexpress the target gene through a constructed expression plasmid named 4D5Fv-PE25. Bacterial growth was monitored periodically during fermentation, and the process was terminated upon reaching the stable phase (Fig. 2A). A deceleration in bacterial growth rate was observed after 8 h of fermentation, indicating that the bacterial growth curve had entered the stationary phase. To minimize the production of impure proteins and to enhance bacterial preparation efficiency and energy savings, bacterial culturing was stopped upon entering the stable growth phase. A yield of 1348.3 g of bacteria was harvested from 55 L of centrifuged fermentation broth. Subsequent to lysis, a total of 229.4 g of purified inclusion bodies was obtained from the bacterial culture.

### Hydrophobic chromatography

Two different types of hydrophobic chromatography gels and their corresponding elution buffers were utilized for the 4D5Fv-PE25. The application of the strong hydrophobic gel Capto Phenyl (HS), failed to completely elute 4D5Fv-PE25 using chromatography buffer A or deionized water. Significant amounts of 4D5Fv-PE25 remained on the gel and could only be eluted using sodium hydroxide (Fig. 2C). On the other hand, the use of the weak hydrophobic gel Capto Butyl, together with the chromatography buffer containing 20% of 30% saturation ammonium sulfate, effectively removes some impurity proteins. However, increasing the ammonium sulfate content to more than 20% diminished the purification effect, while decreasing it below 20% led to the elution of 4D5Fv-PE25 using the equilibrium buffer (Fig. 2B). The elution efficiency of 4D5Fv-PE25 was greatly improved when ethanol was added to the eluent. A chromatography buffer A containing 10% ethanol increased the elution rate to more than 80%, and sodium hydroxide or deionized water could be employed to completely elute the remaining 4D5Fv-PE25 on the Capto Butyl gel (Fig. 2C).

### Ion exchange chromatography

**Fig. 3:**
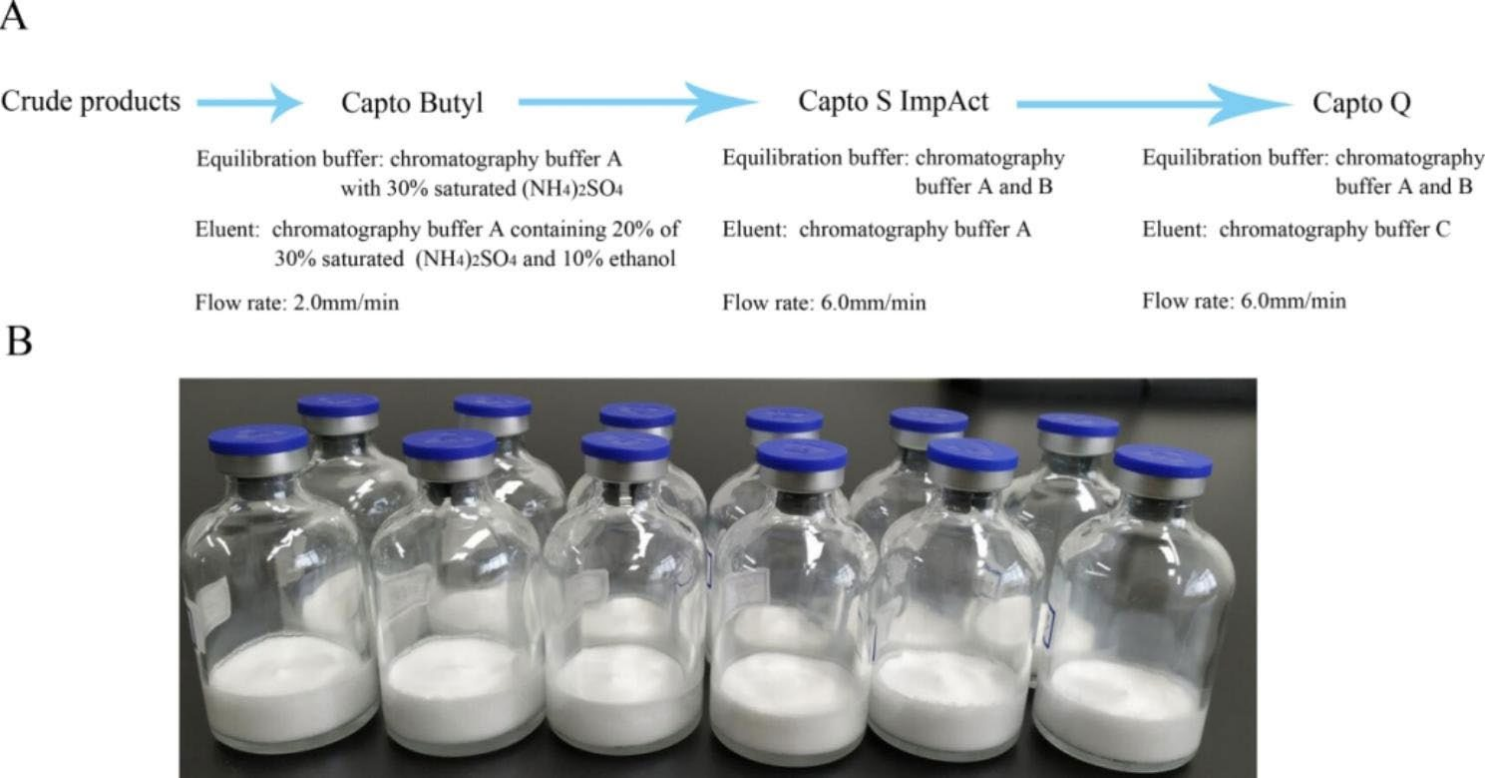
Purification process and freeze-dried products of 4D5Fv-PE25. **A**: Purifying processes of the 4D5Fv-PE25. **B**: Freeze-dried powder of the 4D5Fv-PE25.

The collection started with using Capto S ImpAct to remove the large impurity protein, subsequently, the 4D5Fv-PE25 was collected along with small molecular impurities. Capto Q was then used to remove any remaining small molecular weight impurities and to bind the target protein to the gel. The 4D5Fv-PE25 with sufficient purity was then collected using elution (Fig. 2D). The flow rate of chromatography was found to have an effect on purification. Thus, the flow rate experiment for Capto S ImpAct chromatography was optimized. The findings of the experiment demonstrated that a flow rate of 6.0 mm/min was more effective in removing impurities than a flow rate of 15.0 mm/min (Fig. 2E). Using the optimized condition, the 4D5Fv-PE25 samples were further purified with hydrophobic (Capto Butyl), ion exchange (Capto S ImpAct and Capto Q) resins, resulting in a purity level above 96% (Fig. 2F and G). The chromatography process had a recovery rate of 56.06% (Table 1). The final preparation process of 4D5Fv-PE25 was established (Fig. 3A).

**Table 1:** Protein recovery rate of each step (n = 8)

Process step	Bacteria to inclusion body	Inclusion body to the crude product	Capto Butyl	Capto S ImpAct	Capto Q	Sephadex G25
Protein recovery (%)	17.01	7.34	84.29	81.73	88.15	92.32

### Freeze-dried powder of the 4D5Fv-PE25

Chromatography buffer C was replaced by Phosphate buffered saline (PBS) through Sephadex G25 chromatography, and the products were collected for concentration optimization. After the configuration, filtration sterilization, filling, and freeze-drying processes, the lyophilized powder of the 4D5Fv-PE25 with a loose texture was obtained (Fig. 3B).

### The evaluation of 4D5Fv-PE25 in vitro

**Fig. 4:**
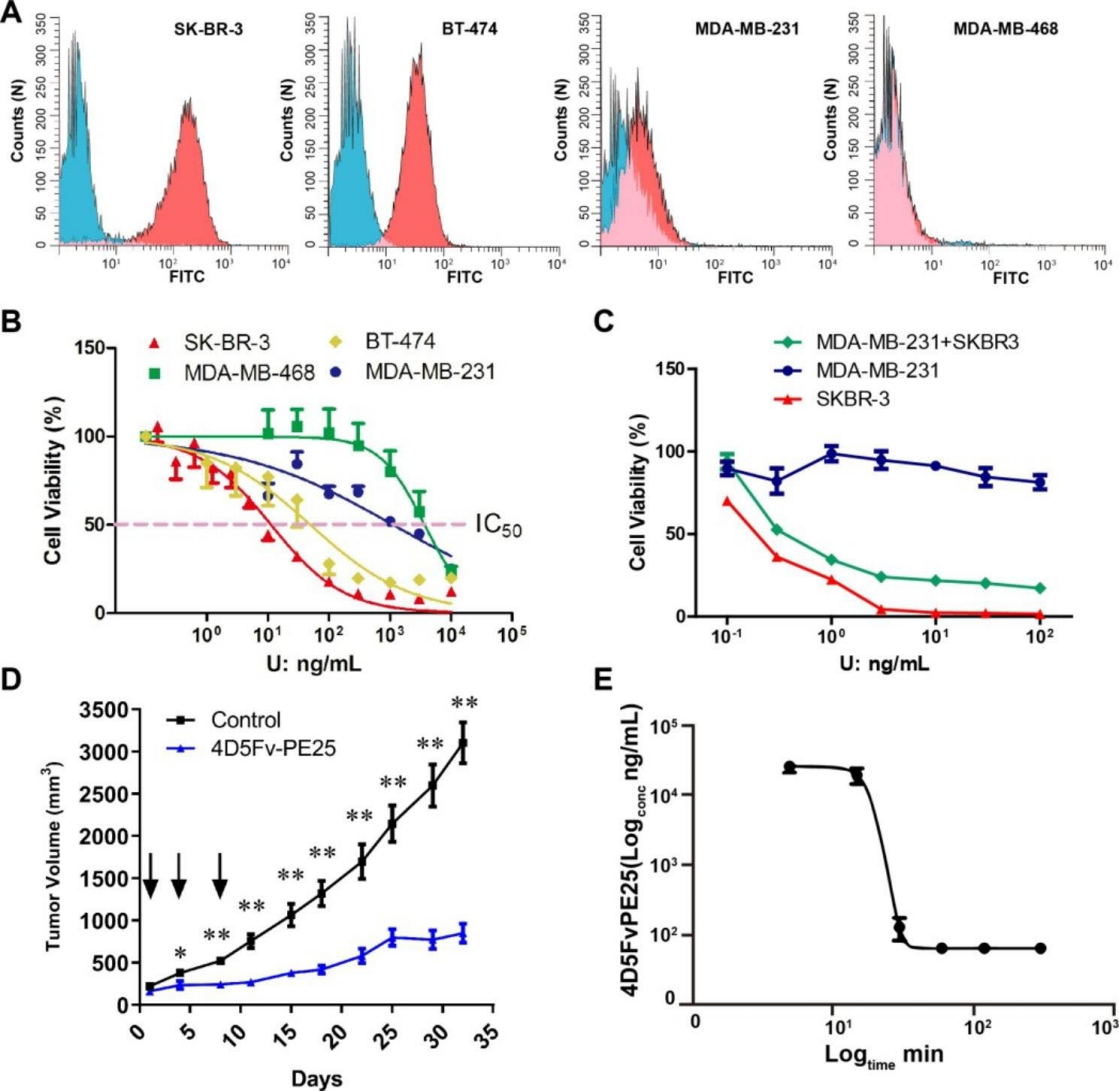
Detection of HER2 expression in breast cancer cell lines and the evaluation of 4D5Fv-PE25 in vitro and in vivo. **A**: Detection of HER2 expression in different cell lines. Blue area: mIgG control, red area: Anti-HER2 antibody. **B**: The killing effect of 4D5Fv-PE25 on different breast cancer cell lines. **C**: The evaluation of the immunotoxin in the co-culture assay. **D**: The inhibition effect on the solid tumor of 4D5Fv-PE25. The black arrows represent the injection time point. n = 5 in control group, n = 10 in 4D5Fv-PE25 group. **E**: Clearance rate of the 4D5Fv-PE25 in mice (n = 6). Data was illustrated as means ± SD, and D was analyzed by two-way ANOVA followed by the Bonferroni test. *P < 0.05, **P < 0.01

Breast cancer cell lines were analyzed for HER2 expression using flow cytometry. SK-BR-3 and BT-474 were used as the positive control due to their high HER2 expressions. SK-BR-3 expressed significantly higher levels of HER2 than BT-474. The triple-negative breast cancer cell lines, MDA-MB-231 and MDA-MB-468, were used as negative controls. MDA-MB-468 had no HER2 expression while HER2 expression in MDA-MB-231 was observed at low levels (Fig. 4A).

IC_50_, a widely used index to investigate the cytotoxic effect of samples, was used to determine the inhibitory effect of 4D5Fv-PE25 on cells. Prior to freeze-drying, the IC_50_ of 4D5Fv-PE25 was 11.29 ng/mL for SK-BR-3, 49.66 ng/mL for BT-474, and more than 1.0 × 10^3^ ng/mL for HER2 negative breast cancer cells (Fig. 4B). In the co-culture condition of SK-BR-3 and MDA-MB-231, 4D5Fv-PE25 showed a partial inhibitory effect on cell proliferation (Fig. 4C). The cytotoxic effect of 4D5Fv-PE25 on breast cancer cells was observed to be positively correlated with the amount of HER2 protein expression. This discovery not only confirmed the inhibitory effect of 4D5Fv-PE25 on breast cancer cells but also demonstrated the specificity of HER2 target recognition.

Freeze-dried powder form effectively prolongs the shelf life of protein drugs, but impacts the drug activity. The 4D5Fv-PE25 powder was reconstituted to determine its bioactivity. The findings demonstrate that the IC_50_ to SK-BR-3 and BT-474 cell lines were 12.53 ng/mL and 53.12 ng/mL, with recovery rates of 86.67% and 86.75% during the freeze-drying process (Table 2).

**Table 2:** Activity of products before and after the freeze-drying process ($\stackrel{-}{X}$+S; n = 8)

Cell line	SK-BR-3	BT-474
IC_50_ before freeze-drying (ng/mL)	11.29 ± 1.76	49.66 ± 6.00
IC_50_ after freeze-drying (ng/mL)	12.79 ± 0.86	56.24 ± 7.04

### Inhibitory effect of the 4D5Fv-PE25 on tumor growth and its catabolism in vivo

HER2-positive xenograft tumor mice were injected with 4D5Fv-PE25 (5 mg/kg) through the tail vein on the 1st, 4th, and 8th day when the tumor volume of mice reached 200 mm^3^. Then the tumor volume of mice was measured and calculated periodically. The tumor volume of mice in the treatment group was significantly reduced than in the untreated group. It showed a significant inhibitory effect on the 4th day after the injection of 4D5Fv-PE25, and the inhibitory effect on solid tumors lasted until the final stage of the observation period (24 days) (Fig. 4D). We then examined the half-life of the 4D5Fv-PE25 by detecting its residue in the plasma of mice after injection. The results indicated the substance only existed for under 60 min inside the body, and the residues decreased sharply from 15 to 30 min after injection (Fig. 4E). It should be noted that the concentration of 4D5Fv-PE25 was calculated based on the residual toxic activity in plasma, and it was not excluded that the decrease of 4D5Fv-PE25 activity in the blood circulation was responsible for such a result. The loss of 4D5Fv-PE25 activity in plasma predicted the disappearance of its killing effect in animals. However, this did not reduce the inhibitory effect and length of 4D5Fv-PE25 in vivo. Overall, 4D5Fv-PE25 showed high potential for the treatment of HER2-positive breast cancer both in vivo and in vitro.

## Discussion

HER2-positive breast cancer has the highest incidence rate in the world, accounting for 7.26% of all new malignant tumors [24]. Early-stage patients have a 30-40% risk of recurrence and metastasis even after standardized and systemic treatment [25], and advanced HER2-positive breast cancer patients have a low 5-year survival rate of 27% [26]. In targeted therapy, the efficiency of drug delivery largely depends on the molecular weight of the drug component [27]. Monoclonal antibodies and ADCs typically have a molecular weight above 150 kDa [28]. Synthetic drugs like ADCs require multiple purification procedures to eliminate extraneous components and raw materials, leading to increased costs and posing a challenge to drug uniformity [29]. Small peptides such as immunotoxin, which are easily modifiable, can be a better alternative [30].

Immunotoxins are targeted drugs obtained via heterologous expression by fusing a targeted vector with a gene fragment of a toxin protein through gene recombination technology. Immunotoxins can be recognized by specific receptors and internalized through receptor-mediated endocytosis. This enables the toxin to be enriched and activated in the target cells, leading to an increase in therapeutic efficiency and a reduction in effects on non-specific cells [31]. This targeted therapy has mild side effects and is beneficial for the treatment of vulnerable patients such as children and the elderly [32]. The 4D5Fv-PE25 was designed based on the antibody variable region of trastuzumab and *Pseudomonas aeruginosa* cytotoxin. The 4D5Fv-PE25 was produced using a highly controllable, low-cost, high-yield, and time-saving prokaryotic expression system [33, 34]. Using *E. coli.* as the expression strain effectively reduced the toxicity of PE25 to the host, while increasing expression efficiency [35]. After high-density cultivation in an 80 L fermentor, the bacteria yielded 24.5 g/L. Prokaryotic inclusion bodies are characterized by impurity proteins [36], making the purification of the target protein the key process in successfully preparing 4D5Fv-PE25. First, ammonium sulfate was used to precipitate the refolding solution and remove the polymers (major impurities). Afterward, hydrophobic chromatography and ion exchange chromatography were carried out for samples, which were not only able to remove a large amount of ammonium sulfate but also purify the protein. After the improving processes, the purity of the final product was more than 96%. Small molecule drugs (< 1.5 kDa) or small proteins (< 40 kDa) can diffuse through tumor tissue, which leads to low accumulation within the tumor [37]. Therapeutic proteins have a molecular weight of around 40–800 kDa and are less likely to diffuse through blood vessels and into the circulatory system [38]. The molecular weight of the 4D5Fv-PE25 is only 52 kDa, which allows it to accumulate in tumors and provides the advantage of being small and easily penetrable. Immunotoxins have higher repeatability between batches and more consistent pharmacokinetic characteristics when compared to ADCs or other synthetic drugs. They are much more efficient, but a lack of suitable manufacturing systems for cost-effective mass production is a current limitation [39–43]. Through the preliminary design and process optimization of the immunotoxin, we were able to solve problems in production and create a low-cost and efficient process to prepare 4D5Fv-PE25.

Subsequently, we assessed the potential of using 4D5Fv-PE25 as a treatment for HER2-positive breast cancer. To determine the appropriate dosage for in vivo and in vitro assays, we conducted a cell survival assay. The IC_50_ of 4D5Fv-PE25 against HER2-positive breast cancer cell line SK-BR-3 was found to be 11.29 ng/mL, significantly lower than the IC_50_ value of 3.00 µg/mL observed for HER2-negative breast cancer cell line MDA-MB-468. This result confirmed that 4D5Fv-PE25 effectively recognizes and inhibits the growth of HER2-positive breast cancer cells. Furthermore, normal cells expressing low levels of HER2 should remain unaffected by this toxin [44], indicating that 4D5Fv-PE25 is safe for healthy tissue. Following freeze-drying, the IC_50_ of the 4D5Fv-PE25 can still reach 12.53 ng/mL with an 89% activity recovery rate. In comparison to monoclonal antibodies and other small-molecule drugs, 4D5Fv-PE25 demonstrates significant anticancer activity [45]. According to recent studies [46], up to 90% of new drugs fail to demonstrate effectiveness in animal models. Consequently, in vivo experimentation is an essential step in assessing the potential effects of drugs. We selected breast cancer xenograft tumor mice expressing high levels of humanized HER2 as a model for HER2-positive breast cancer, and in vivo experimentation with 4D5Fv-PE25 exhibited a potent and long-term inhibitory effect on the solid tumor. Efficacy was maintained for over 24 days following three injections in mice. However, kinetic experiments revealed that the activity of 4D5Fv-PE25 can be entirely disappeared from the mouse body within 1 h of administration. Although it cannot be concluded that 4D5Fv-PE25 has been entirely cleared in mice, its half-life may be short, which needs to be further confirmed by experiments. A shorter half-life means a shorter contact time between the drug and blood vessels, which effectively reduces the occurrence of vascular leakage syndrome (VLS), a common side effect in cancer treatment [47, 48]. These results indicate that the 4D5Fv-PE25 has the characteristics of fast clearance in the body and a long-time effect, which is not contrary to common sense. Many good anti-cancer drugs (Paclitaxel et al.) in the market have such characteristics [49, 50]. This may be due to the direct killing effect caused by the enrichment of 4D5Fv-PE25 in tumor tissues, as well as special biological effects, such as the induction of immune cell infiltration to generate additional anti-tumor immune responses [51]. Of course, this needs to be verified by a large number of experiments, which is also a very worthy research direction.

## Conclusion

In summary, we designed and manufactured a lyophilized powder immunotoxin 4D5Fv-PE25 with stable purity above 96% for HER2-positive breast cancer. The preparation process of 4D5Fv-PE25 has the advantages of good controllability, high preparation efficiency, and low-quality risk, which is conducive to industrialization. Our in vivo and in vitro studies confirmed the significant targeted recognition and inhibition of 4D5Fv-PE25 to HER2-positive breast cancer cell lines and solid tumors. Mouse experiments further showed that it had a long-term inhibitory effect on tumors despite the rapid loss of 4D5Fv-PE25 activity in plasma, providing perspective for future mechanistic exploration of 4D5Fv-PE25. Overall, our findings suggest that 4D5Fv-PE25 is a promising candidate drug for targeted therapy in HER2-positive breast cancer, given its high development value.

## Methods

### Design of the immunotoxins

The plasmids containing the VL, G_4_S, VH, and Linker, Furin cleavage site, PE25, KDEL sequence were synthesized by Songon Biotech Company. The target gene sequence was digested by the *EcoR I* and *Spe I* (Takara Bio) restriction endonucleases, and then, T4 ligase (Takara Bio) was used to connect it to a new plasmid. The 4D5Fv-PE25 sequence was inserted into the pET-28a(+) vector(Novagen, Madison, WI, USA)using the *Xho I*/*Xba I* (Takara Bio) restriction enzyme sites. The recombinant plasmid was transformed into BL21 (DE3) *E. coli*.

### Bacteria amplification

The 4D5Fv-PE25 strain (BL21 (DE3)) was inoculated onto a solid Luria-Bertani (LB) medium plate containing 50 µg/mL kanamycin (Kan^+^) and cultured at overnight 37℃. A single clone was selected and inoculated in 120 mL liquid LB medium (Kan^+^), cultured at 37℃ for 6 h. Then the culture medium was transferred into a 6,000 mL LB medium (Kan^+^) and continued to culture for 10 h.

### Fermentation and expression

The seed liquid was inoculated into 50 L M9 medium (tryptone 9.63 g/L, yeast extract 4.81 g/L, (NH4)_2_SO_4_ 1.21 g/L, K_2_HPO_4_•3H_2_O 12.07 g/L, KH_2_PO_4_ 1.60 g/L, VitB_1_ 0.07 g/L, glucose 27.78 g/L, MgSO_4_•7H_2_O 1.11 g/L, pH 7.2) cultured in the fermentation tank (NEW BRUNSWICK BF-5000), and added 15 mL of trace element mother liquor (FeCl_3_•6H_2_O 5.0 mg/mL, CoCl_2_•6H_2_O 0.5 mg/mL, ZnSO4•7H_2_O 1.5 mg/mL; MnSO_4_•H_2_O 0.3 mg/mL, CuSO_4_•5H_2_O 1.5 mg/mL). It was incubated for 155 min at 37℃, 220 rpm, and 100% dissolved oxygen, then added 2,400 mL glucose supplement (glucose 0.5 g/mL) and 1,500 mL LB supplement (tryptone 0.08 g/mL, yeast extract 0.04 g/mL, NaCl 0.02 g/mL), continued to incubate for 30 min. Afterward, Isopropyl-beta-D-thiogalactopyranoside (IPTG) was added to a final concentration of 0.1 mM to induce the 4D5Fv-PE25 expression, and ammonia was used for adjusting pH to 6.90–7.20 during the fermentation process. The absorbance (OD_600_) of the fermentation broth was measured by random sampling. The fermentation broth was collected after 280 min of induced expression, and the pelleted bacteria were collected using a continuous flow centrifuge at 7,227 g, 10℃.

### Preparation of crude products

The pelleted bacteria were washed with Tris-EDTA (TE) buffer (100 mM Tris-HCl, 10 mM ethylene diamine tetraacetic acid (EDTA)). Then use the cell crusher to crush the bacteria and collect the bacteria pellet. The pellet was washed successively with lysis bufferI(50 mM Tris-HCl, 20 mM EDTA, 100 mM NaCl, 5% Triton-X 100, pH 8.0), lysis bufferII(50 mM Tris-HCl, 20 mM EDTA, 100 mM NaCl, pH 8.0) and 2 M guanidine-HCl buffer (2 M Guanidine-HCl 100 mM Tris-HCl, 50 mM NaCl, 6 mM dithiothreitol (DTT), pH 8.0) at the ratio of 10 mL/mg, the clean 4D5Fv-PE25 inclusion bodies were obtained finally. The denature buffer (100 mM sodium phosphate, 6 M Guanidine-HCl, 2 mM EDTA, 6 mM DTT, pH 8.0) was added into 4D5Fv-PE25 inclusion bodies at a ratio of 12 mL/mg for denaturation, then centrifuged at 16,260 g for 20 min to collect the supernatant. The denatured solutions were dropwise added into refolding buffer (100 mM sodium phosphate, 1.4 mM glutathione (GSH), 1 mM EDTA, 0.5 M Urea, 0.5 M Arginine, pH 8.5), standing for 12 h at 10℃. Pre-cooled ammonium sulfate solid was added to 30% saturation and stored overnight at 4℃. The 4D5Fv-PE25 crude products containing ammonium sulfate were obtained by centrifugation at 16,260 g, 4℃ for 30 min.

### Refining processes

#### Hydrophobic chromatography

Hydrophobic chromatography was implemented using the protein purification system from GE Healthcare (ÄKTA pure 150 Convenience) with columns consisting of strong hydrophobic gel Capto Phenyl (HS) and weak hydrophobic gel Capto Butyl (GE Healthcare).

Chromatography buffer A (20 mM sodium phosphate, 1 mM EDTA, pH 6.5) with 30% saturation ammonium sulfate was used to equilibrate the gel column (GE XK 26/40) with 5 column volumes, then the crude products were loaded at a flow rate of 2.0 mm/min. The effects of gradient elution (chromatography buffer A with 10%, 20%, 30%, 40%, 50%, 60%, 70%, 100% (v/v) of 30% saturation ammonium sulfate) and eluent containing ethanol (1%, 5%, 10% (v/v)) were compared. The Phenyl/Butyl columns were eluted with water, followed by 0.5 M NaOH. Finally, the eluates were analyzed to assess the purification efficiency of the hydrophobic chromatography by 12% polyacrylamide gel electrophoresis (SDS-PAGE).

#### Ion exchange chromatography

The chromatographic column GE XK 16/40 and GE XK 26/40 were separately filled with Capto S ImpAct (GE Healthcare) gel and Capto Q (GE Healthcare) gel. 5 column volumes of chromatography buffer A and chromatography buffer B (20 mM sodium phosphate, 1 mM EDTA, 1 M NaCl, pH 6.5) were used respectively for regeneration. The hydrophobic chromatographic eluent was separately sampled on Capto S ImpAct and Capto Q gel for purification. Capto S ImpAct was sampled at 6.0 mm/min and 15.0 mm/min, the column was equilibrated with chromatography buffer A after loading, and the flow-through liquid was collected to detect the purity. The 4D5Fv-PE25 on Capto Q was eluted with chromatography buffer C (20 mM sodium phosphate, 1.0 mM EDTA, 10% NaCl, pH 6.5), and elution peaks were collected to detect protein purity by SDS-PAGE.

#### Filtration chromatography and protein purity detection

GE XK 26/100 column was filled with Sephadex G25 (GE Healthcare) gel and equilibrated with 5-column volumes of phosphate buffer saline (PBS). The elution peaks were collected after loading the sample. The high-resolution gel filtration column Superdex 75 10/300 GL (GE Healthcare) was used to detect purity with a flow rate of 6.5 mm/min, and the wavelength for UV detection was 280 nm.

### Freeze-dried process

The 4D5Fv-PE25 was prepared according to the dosage of 10mL products (containing 300 mg 4D5Fv-PE25, 0.35% (w/w) human albumin, 4% (w/w) mannitol) in the 50 mL vial. The semi-products were filtered by the 0.22 µM filter membrane, then filled and lyophilized by a freeze-drying machine (Christ Epsilon 60D Lscplus).

### Cell culture

The human breast cancer cell lines MDA-MB-231 (ATCC HTB-26), MDA-MB-468 (ATCC HTB-132), BT-474 (ATCC HTB-20), and SK-BR-3 (ATCC HTB-30) were cultured in DMEM medium with 10% fetal serum, at 37℃ and 5% CO_2_. The cells were detached using trypsin when they reached the logarithmic growth phase to prepare cell suspensions for future use.

### Detection of HER2 expression

Breast cancer cells were blocked with 5% rabbit serum (Cedarlane, CL1000) in PBS for 20 min, then added 2.5 µg mIgG (Sigma, I5381) or an anti-HER2 antibody (R&D, MAB1129). FITC (fluorescein isothiocyanate) goat anti-mouse IgG (0.25 µg in 100 µL IF buffer; Cedarlane, CLCC30001) was added and incubated for 30 min, followed by a washing step with IF buffer. Finally, t the sample was fixed with 4% paraformaldehyde (PFA) and measured using flow cytometry.

### Cytotoxicity assays

The 4D5Fv-PE25, both before and after lyophilization, were prepared as a 1 mg/mL solution. Four different types of cell lines (including MDA-MB-231, MDA-MB-468, BT-474, and SK-BR-3 cell lines) were seeded at a density of 8,000 cells per well in 96-well plates. A co-culture experiment was conducted by seeding SK-BR-3 and MDA-MB-231 cell lines, each with a density of 4,000 cells, into the same well. The 4D5Fv-PE25 solution was added with the final concentration of 1.0 × 10^4^, 3.0 × 10^3^, 1.0 × 10^3^, 3.0 × 10^2^, 1.0 × 10^2^, 3.0 × 10^1^, 1.0 × 10^1^, 3.0, 1.0, 3.0 × 10^− 1^, 1.0 × 10^− 1^ ng/mL, and determined the cell viability according to Juliette Sauveur’ method after 72 h culture [52].

### In vivo studies

The solution of 4D5Fv-PE25 utilized in the experiments involving mice was a freeze-dried product redissolved in water for injection (WFI). The initial concentration was 1 mg/mL, and the sample was diluted to the appropriate concentration. The control group was injected with the lyophilization protective agent.

BT-474 xenograft mice (based on C-NKG immunodeficient mice, obtained from Cyagen Biosciences) with high expression of humanized HER2 were used as animal models. Upon reaching a tumor volume of 200 mm^3^, the 4D5Fv-PE25 (5 mg/kg) was injected via the tail vein on the 1st, 4th, and 8th day, and the tumor volume was monitored for 24 days after the final injection. Tumor dimensions of mice were measured using calipers, and their volumes were calculated using the formula: Volume = 4/3 × π × length × width×depth/8 [44].

#### Residue analysis

C57BL/6 mice were injected with 5 mg/kg 4D5Fv-PE25, and 25 µL blood samples were collected at 5, 15, 30, 60, 120, and 300 min after injection. The blood samples were collected in EDTA coated centrifuge tube and plasma was collected after centrifuging at 2,000 g for 3 min. The 4D5Fv-PE25 samples were prepared at the concentration of 1.0 × 10^4^, 3.0 × 10^3^, 1.0 × 10^3^, 3.0 × 10^2^, 1.0 × 10^2^, 3.0 × 10^1^, 1.0 × 10^1^, 3.0, 1.0, 3.0 × 10^− 1^, 1.0 × 10^− 1^ ng/mL and plasma sample were diluted 10 times. The samples were added to 96-well plates containing 1.5 × 10^4^ SK-BR-3 cells (200 µL) per well and cultured at 37℃ for 2 days. Added 10 µL 50 µCi/mL 3 H-Thymidine to each well, and recorded the number of ray counts per minute (CPM) [44, 53]. The CPM values and concentration of the 4D5Fv-PE25 group were used to make the standard curve and then calculated the relative concentration of 4D5Fv-PE25 in plasma samples from the CPM values measured in the experimental group.

### Statistical analysis

Data were analyzed by GraphPad Prism 5.0 software. The data in the curve were presented as means (Ā) ± standard deviation (SD), two way ANOVA was performed to determine the difference between the data. and differences were considered statistically significant when p < 0.05. The software Image J version 1.48 was used to roughly calculate the purity of protein by gray analysis.

The recovery was calculated by the following formula.

$${\rm{Recovery}}\,(\% )\,{\rm{ = }}\,\frac{{{{\rm{C}}_{aft}} \times {{\rm{V}}_{aft}}}}{{{{\rm{C}}_{bef}} \times {{\rm{V}}_{bef}}}}\, \times {\rm{100}}$$

C_*aft*_ represents the protein concentration after treatment, V_*aft*_ represents the sample volume after treatment, C_*bef*_ represents the protein concentration before treatment, V_*bef*_ represents the sample volume before treatment. The concentration of protein was measured by the Bradford assay [54].

The purity of the sample was calculated by the formula:

$${\rm{Purity}}\,{\rm{(\% )}}\,{\rm{ = }}\,\frac{{{{\rm{A}}_{tar}}}}{{{{\rm{A}}_{tar}}{\rm{ + }}{{\rm{A}}_{mis}}}}\,{\rm{ \times 100}}$$

A_*tar*_ represents the peak area of the target protein and A_*mis*_ represents the peak area of miscellaneous protein.

## Data Availability

All data are contained within the article.
